# Multidrug-Resistant *Candida auris* and its Role in Carcinogenesis: A Scoping Review

**DOI:** 10.21315/mjms-09-2024-691

**Published:** 2025-02-28

**Authors:** Wan NurHazirah Wan Ahmad Kamil, Mukarramah Zainal, H.M.H.N. Bandara, Mohd Hafiz Arzmi

**Affiliations:** 1 Department of Fundamental Dental and Medical Sciences, Kulliyyah of Dentistry, International Islamic University Malaysia, Kuantan, Pahang, Malaysia; 2 Cluster of Cancer Research Initiative, Kulliyyah of Dentistry, International Islamic University Malaysia, Kuantan, Pahang, Malaysia; 3 Centre of Oral Maxillofacial Diagnostics and Medicine Studies, Faculty of Dentistry, Universiti Teknologi MARA, Sungai Buloh, Selangor, Malaysia; 4 Centre of Preclinical Science Studies, Faculty of Dentistry, Universiti Teknologi MARA, Sungai Buloh, Selangor, Malaysia; 5 Bristol Dental School, University of Bristol, Bristol, United Kingdom; 6 Melbourne Dental School, The University of Melbourne, Victoria, Australia

**Keywords:** Candida auris, multidrug resistance, nosocomial infection, risk factors, cancer, carcinogenesis

## Abstract

*Candida auris* was listed as a critical fungal priority group pathogen by the World Health Organization (WHO) in 2022. It has become a leading cause of invasive candidiasis in serious nosocomial infections globally. While *Candida* species, particularly *C. albicans*, are linked to cancer development, the role of *C. auris* in carcinogenesis remains unexplored. This scoping review aimed to evaluate the existing evidence on the role of *C. auris* infection in carcinogenesis and its associated risk factors. Following the PRISMA-ScR guidelines, a comprehensive search of three databases was conducted from January 2003 to January 2024 to identify studies addressing the role of *C. auris* infection in cancer development and its associated risk factors. A total of 124 articles were identified, of which six met the inclusion criteria. These studies reported the risk factors associated with *C. auris* infection in cancer patients. The findings showed an increased susceptibility of cancer patients to *C. auris* infections. However, to date, no direct relationship has been reported between *C. auris* infection and cancer development due to the limited accuracy of diagnostic tools. In conclusion, *C. auris* infections increase the susceptibility of cancer patients but are not directly involved in carcinogenesis, indicating the urgency for an accurate diagnostic tool for *C. auris* detection and specialised infection-control measures for cancer patients.

## Introduction

Since 2022, *Candida auris* (*C. auris*) has been categorised as a critical group of fungal pathogens by the World Health Organization (WHO) due to its ability to cause invasive candidiasis in healthcare settings (1). It was first identified in 2009 in over 40 countries, with alarming morbidity and mortality rates, especially in immunocompromised individuals (2). *C. auris* infection has been reported in several Asian countries, including Malaysia and Singapore. In Malaysia, the first fatal case of *C. auris* infections was reported in a neutropenia patient (3).

Epidemiological studies revealed that *C. auris* infections are primarily associated with healthcare facilities, particularly intensive care units, long-term care facilities, and hospitals (4). The overall mortality of invasive candidiasis with *C. auris* ranged from 29% to 53% (1). Cancer patients are among the populations at a higher risk of *C. auris* infections due to their compromised immune systems and frequent exposure to healthcare environments, making them susceptible to infections (2, 5). The pathogen can be easily transmitted from patient to patient, potentially contaminating the healthcare environment and leading to outbreaks (6, 7).

Cancer is the leading cause of death worldwide, posing a threat to life expectancy in every country regardless of the level of economic development (8). According to GLOBOCAN 2022, an incidence of 19,976,499 cancer cases have been reported, with 9,743,832 deaths in 2022 (9). As the disease advances, uncontrolled cell growth and tissue invasion occur, leading to distant metastases. Metastases are the main cause of cancer-related death (9). This disease advancement promotes the shedding of malignant cells from the primary site tumour to distant organs via the bloodstream, where they attach and grow, mimicking the behaviour of the primary tumour (9).

Recent studies have suggested a connection between yeast infection and an increased risk of developing certain types of cancer (10, 11). These infections are reportedly involved in cancer development, such as cancer initiation, establishment, and spread (12, 13). *Candida* infections are often associated with a weakened immune system. Individuals with compromised immune systems, such as those undergoing cancer treatment or HIV/AIDS patients, are susceptible to *Candida* infections, including *C. auris*, which is common in cancer patients, especially those undergoing chemotherapy (14, 15).

Given the growing threat of *C. auris* and its potential impact on cancer patients, it is crucial to understand the current epidemiology, risk factors, clinical manifestations, and management strategies for this emerging fungal pathogen. The association between *C. auris* infections and cancer involves various aspects, including clinical, epidemiological, and microbiological. However, the role of *C. auris* in carcinogenesis remains unclear. This scoping review aims to elucidate the association between *C. auris* infections and cancer, synthesising findings from various studies highlighting the epidemiology, clinical characteristics, and risk factors of *C. auris* infections in cancer patients.

## Methods

### Data Sources

This review was conducted according to the Preferred Reporting Items for Systematic Reviews and Meta-Analyses extension for Scoping Reviews (PRISMA-ScR) guidelines (16, 17). The literature review was based on a search of PubMed, Scopus, and Web of Science databases for articles dealing with *in vitro*, *in vivo*, *ex vivo* trials, case reports, retrospective studies, and observational studies reporting the association of *C. auris* infection in carcinogenesis, dated between January 2003 to January 2024. The following search string was applied to the databases: “(*Candida auris* or “*C. auris*”) and (cancer* OR carcinogenesis OR oncogenesis)” [PubMed] and “(*Candida auris*) and (infection)” [Scopus and Web of Science].

### Inclusion Criteria

The works included in the scoping review include:

- Papers dealing with *in vitro*, *in vivo*, and *ex vivo* trials reporting *C. auris* infection associated with carcinogenesis.
- Retrospective analyses, observational studies, and case reports related to *C. auris* infection in cancer patients.

### Exclusion Criteria

The works excluded from the scoping review include:

- Reviews, book chapters, and letters to editors.
- Articles discussing *Candida* infection without *C. auris*.
- Non-English articles.

### Selection of Articles

**Figure 1:**
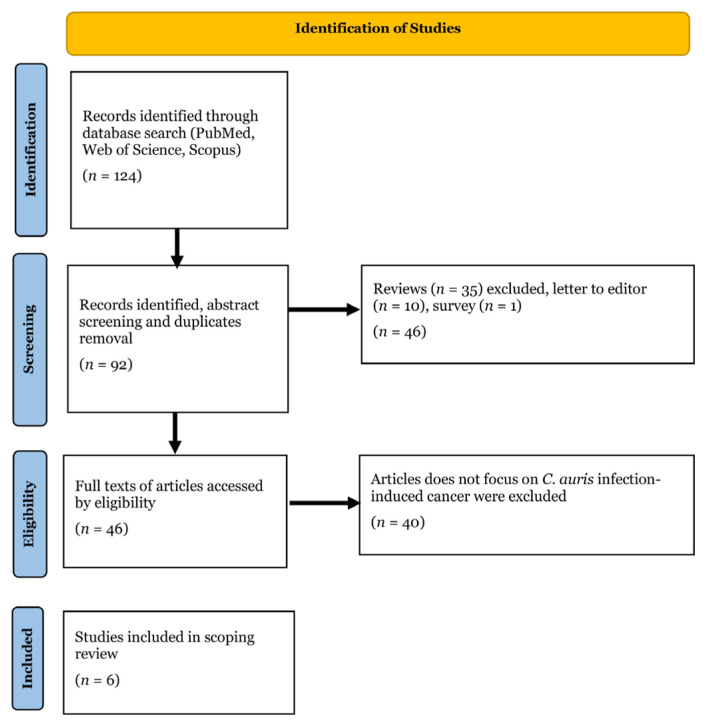
Flowchart illustrating the process following the PRISMA Sc-R guidelines

The electronic literature searches in PubMed, Scopus, and Web of Science databases resulted in 124 articles. Three independent reviewers (WNH, MZ, and MHA) were unanimous regarding the literature selection process. After applying the exclusion and meeting the inclusion criteria, only six articles were included in the scoping review. The selection process of the articles is demonstrated in Figure 1. The analysis of the articles, including an overview of the epidemiology study design, types of cancer, and main findings of the articles, is summarised in Table 1.

## Results and Discussion

### Epidemiological Findings and Risk Factors Associated with C. auris Infection

Over 400,000 bloodstream infections per year have been attributed to *Candida* spp., which are the most common fungi in hospital settings worldwide (4, 18). *C. albicans* is the primary pathogen responsible for candidiasis (25). However, *C. auris* has been reported in over 25 countries on five continents, causing fungemia outbreaks with crude mortality rates varying from 32% to 66% (26–28). Since *Candida* spp. are highly heterogeneous, *C. auris* differs markedly from common and well-studied pathogenic *Candida* spp., such as *C. albicans* and *C. glabrata* (29, 30)*. C. auris* can persistently colonise the host skin, making it easily transmissible between patients (24).

**Figure 2:**
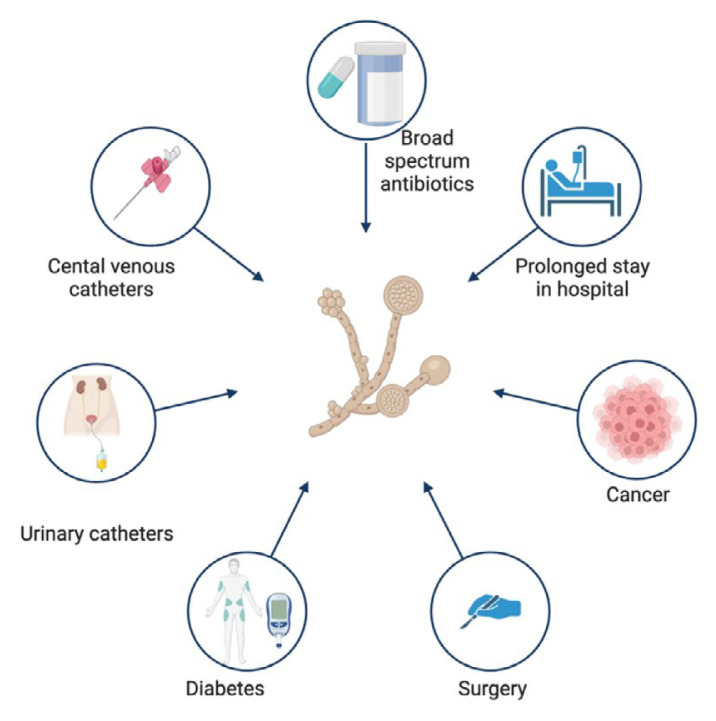
Risk factors associated with *Candida auris* infection

*C. auris* is an opportunistic pathogen that has gained attention due to its ability to cause severe infections and outbreaks in healthcare settings. This yeast can cause life-threatening infections, particularly in individuals with a compromised immune system (30). The risk factors for contracting *C. auris* infection are similar to those for contracting bloodstream infections (BSIs) (Figure 2). These factors include the use of invasive medical devices, such as central venous or urinary catheters, broad-spectrum antibiotic therapy, prolonged hospitalisations, immunosuppressive therapies, the elderly, and major surgical procedures (31, 32).

Patients with cancer are vulnerable and at a higher risk for *C. auris* infections due to several factors, including the malignancy itself and the immunosuppressive treatments they undergo. Treatments such as chemotherapy, radiation therapy, and immunosuppressive agents further compromise immune function, thus reducing the ability to overcome infections (4). The ability of *C. auris* to adhere to medical devices and form biofilms causes its spread and persistence in healthcare settings (6, 33). Biofilms have been found in 90% of catheter-associated infections, indicating the impact that *C. auris* can have in a hospital setting where catheter infections are the leading cause of morbidity and mortality (21).

A retrospective study emphasises the prevalence of *C. auris* infections in adult cancer patients, particularly those with cholangiocarcinoma, prostate cancer, multiple myeloma, Hodgkin’s lymphoma, and colorectal carcinoma with metastases (18). The study highlights that *C. auris* colonisation is common among cancer patients with comorbid conditions, such as diabetes and gastrointestinal or liver diseases (18). The findings emphasise the need to properly screen and monitor *C. auris* in this vulnerable patient population. Other studies have also discussed the types of cancers associated with *C. auris* infections, including colorectal, brain, and pancreatic carcinoma (18–23).

Few case reports have shown that patients tend to develop *C. auris* infection during long-term hospitalisation and with extensive use of broad-spectrum antibiotics (20–22). These cases highlight the increased susceptibility of immunocompromised cancer patients to *C. auris* infection due to their weakened immune systems and frequent hospitalisations (21). This is due to the characteristics of *C. auris*, which can colonise multiple body sites, including the axilla, groin, oral cavity, or the bloodstream, as observed in patients with central venous catheters and those undergoing intensive treatments (34). It can colonise hosts within days to weeks of exposure, and invasive infections may occur within days to months of colonisation (30). *C. auris* is an opportunistic pathogen that inhabits the skin and causes systemic infections in hospital environments, particularly among patients with underlying medical conditions, including those who have contracted COVID-19 (35).

Only one study reported in paediatric patients that *C. auris* bloodstream infections occurred in children with haematological malignancies (19). The incidence of *C. auris* bloodstream infections in the paediatric group was due to the susceptibility of paediatric cancer patients to fungal infections. Strict adherence to infection-control protocols, including contact precautions, hand hygiene, and environmental cleaning, is essential to prevent the spread of *C. auris* in paediatric oncology wards. Healthcare facilities should also consider separating infected patients into separate wards to minimise transmission.

### C. auris and Carcinogenesis

Biofilm formation, phenotypic switching, secretion of lytic enzymes, and high-stress tolerance are the virulence factors of *C. auris* that contribute to nosocomial infection. In addition, *C. auris* can persistently colonise healthcare environments and human hosts despite the reduced adhesins in its genome (36). However, the role of *C. auris* in cancer development remains unclear.

Most of the articles examined in this review did not demonstrate a direct link between *C. auris* and cancer development; rather, cancer patients are more prone to be infected by the species. The true occurrence of *C. auris* candidemia remains poorly defined due to the failure of conventional methods to identify the species (31). Furthermore, the species has also been reported to be wrongly diagnosed with *Candida haemulonii* (37). Thus, these findings highlight the importance of accurately detecting *C. auris* to prevent its spread, particularly among immunocompromised cancer patients.

Regarding the methods of identifying *C. auris* infection, a study mentioned that the yeast is difficult to identify accurately due to the lack of proper diagnostic tools (19). Some studies emphasised the need for rapid and accurate identification of *C. auris* to prevent nosocomial infections and outbreaks. Thus, infection-control measures are needed to manage the spread in healthcare settings (19, 21).

*Candida* spp. including *C. auris*, have the potential to initiate and promote the progression of cancerous processes; however, they are not considered causative agents of cancer (38–42). Candidiasis can occur due to the existing cancer and can be used to predict the cancer severity. Their development may be favoured by immunosuppression resulting from cancer chemotherapy. *Candida* infections have been reported to promote cancer progression by affecting the host via various mechanisms (43):

- Perturbations in the DNA-damage response in host cells cause genetic mutations that accumulate inside the cell, modifying the oncogene expression involved in cell survival and proliferation.
- Oncogenic inflammation in the host cells induced by DNA-damaging fungal toxins and their carcinogenic-inducing metabolites.
- Fungal colonisation or infection results in intense inflammation, favouring the growth of primary tumours and metastases, making tumours resistant to chemotherapy drugs and suppressing the host’s anti-cancer immune responses.

*Candida* spp. has been reported to be isolated from 75% of individuals diagnosed with oral squamous cell carcinoma (OSCC) (44). The majority of *Candida* isolates were *C. albicans*. In addition, oral candidiasis has been observed in patients with haematopoietic neoplasms, head and neck malignancies, and those undergoing chemotherapy or radiotherapy, with a prevalence ranging from 7% to 52%. (45, 46). Animal studies have suggested that infections caused by *C. albicans* can lead to carcinogenesis, similar to other known carcinogenic substances (10, 47, 48). Several studies have also demonstrated a strong correlation between candidiasis and dysplasia in the oral cavity, precancerous disorders, and OSCC (49, 50).

*Candida* promotes carcinogenesis by producing carcinogens, pro-carcinogen metabolism, and other molecular mechanisms (51). A study performed on Sprague-Dawley rats found that an imbalance in the oral microbiome can cause *Candida* hyphal invasion, producing and releasing nitrosamines that can promote the growth and progression of oral cancers (52, 53). These results align with the previous research that reported on the ability of *C. albicans* to catalyse carcinogenesis in the tongues of rats and mice when exposed to repeated applications of nitroquinoline (4-nitroquinoline1-oxide; 4-NQO), similar to the human head and neck cancer (51). In addition, the upregulation of Ki-67, P53, and COX-2 in host cells following infection with *Candida* implies the potential role of this fungus in the malignant transformation of host cells (51, 54).

Furthermore, the upregulation of cell proliferation markers (Ki-67 and P53) has been extensively documented in several malignant conditions (48). The expression of COX-2, an inflammatory marker that converts arachidonic acid to prostanoids (including prostaglandins, thromboxane, and prostacyclin), has been observed in various cancers and precancerous abnormalities, indicating its potential role in promoting cell growth, tumour invasion, and cell death (48). Even though the evidence indicating a direct link of *C. auris* to carcinogenesis is still limited, however, the evidence on the role of *Candida* in cancer development may hypothesise a similar pathway for this emerging fungal pathogen toward cancer initiation and progression (48, 55, 56).

## Conclusion

Although evidence directly linking *C. auris* to cancer development is currently lacking, the increased susceptibility of cancer patients to *C. auris* infections may indicate the link to cancer initiation and progression. This evidence also emphasises the need for enhanced and improved treatment of *C. auris* infection in cancer patients. Nevertheless, further research is essential to elucidate the mechanisms of *C. auris* and its impact on cancer progression. By addressing these challenges, healthcare providers can better manage *C. auris* infections and improve outcomes for cancer patients.

## Figures and Tables

**Table 1:** Overview of the articles that were selected in this scoping review

Authors	Title	Study design	Type of cancer	Main findings
Khan et al. (18)	Invasive *Candida auris* infections in Kuwait hospitals: epidemiology, antifungal treatment and outcome	Retrospective study	Seven cases: Cholangiocarcinoma (*n* = 2) Cancer prostate (*n* = 1) Multiple myeloma (*n* = 1) Hodgkin’s lymphoma (*n* = 2) Colorectal carcinoma with metastases (*n* = 1)	Six patients were colonised with *C. auris* at one or more body sites The *C. auris* infections were mainly found in adult patients with cancer, diabetes, gastrointestinal/liver diseases, and extended hospital stays. The true incidence of *C. auris* candidemia, however, remains poorly defined because of the failure of conventional identification methods to identify *C. auris* accurately.
Berrio et al. (19)	Bloodstream infections with *Candida auris* among children in Colombia: clinical characteristics and outcomes of 34 cases	Retrospective study	Haematological malignancies (*n* = 4)	12% of the paediatric patients with *C. auris* bloodstream infections had cancer as an underlying condition The report emphasises the need for rapid and accurate identification of *C. auris* and infection-control measures to prevent its spread.
Noginskiy et al. (20)	A case of multiple myeloma presenting as *Streptococcus pneumoniae* meningitis with *Candida auris* fungemia	Case report	Multiple myeloma (*n* = 1)	The paper discusses a case of multiple myeloma in which the patient developed *C. auris* during their hospital stay. Therefore, there is a correlation between *C. auris* and cancer, specifically in patients with weakened immune systems due to cancer.
Teke et al. (21)	The second case of *Candida auris* candidemia from Turkey: an impending threat to the global health	Case report	Brain cancer (*n* = 1)	The underlying immunodeficiency due to multiple myeloma, made them susceptible to severe bacterial and fungal infections.
Meena et al. (22)	*Candida auris* emergence in the Himalayan foothills: first case report from Uttarakhand, India	Case report	Pancreatic carcinoma (*n* = 1)	37/female underwent Whipple procedure for pancreatic carcinoma Blood culture showed positive for *C. auris* infection due to prolonged surgery, central venous catheter, broad-spectrum antibiotics, and prolonged hospital stay.
Bhattacharya et al. (23)	*Candida auris* infection among patients with cancer in an oncology centre in Eastern India	Retrospective study	Haematological malignancies (*n* = 3)	*C. auris* strains were identified by Sanger-based DNA sequencing of the internal transcriber spacer (ITS) gene. 11 cases of *C. auris* infections (8 from patients with solid-organ tumours and three from haematological malignancy) were detected.
